# Three novel mutations in KIF21A highlight the importance of the third coiled-coil stalk domain in the etiology of CFEOM1

**DOI:** 10.1186/1471-2156-8-26

**Published:** 2007-05-18

**Authors:** Wai-Man Chan, Caroline Andrews, Laryssa Dragan, Douglas Fredrick, Linlea Armstrong, Christopher Lyons, Michael T Geraghty, David G Hunter, Ahmad Yazdani, Elias I Traboulsi, Jan WR Pott, Nicholas J Gutowski, Sian Ellard, Elizabeth Young, Frank Hanisch, Feray Koc, Bruce Schnall, Elizabeth C Engle

**Affiliations:** 1 Program in Genomics, Children's Hospital Boston, 300 Longwood Ave, Boston, MA, 02115, USA; 2 Department of Neurology, Children's Hospital Boston, 300 Longwood Ave, Boston, MA, 02115, USA; 3 Harvard Medical School, 25 Shattuck Street, Boston, MA, 02115, USA; 4 Department of Ophthalmology, Colorado Permanente Group, 10350 East Dakota Avenue, Denver, CO, 80231, USA; 5 Department of Ophthalmology, University of California, San Francisco, 10 Koret Street, Room K335, San Francisco, CA 94143-0730, USA; 6 Children's and Women's Health Centre of British Columbia, Provincial Medical Genetics Programme, 4500 Oak Street, Vancouver, BC, V6H 3N1, Canada; 7 Department of Ophthalmology, University of British Columbia and British Columbia Children's Hospital, 4480 Oak Street, Vancouver, V6H 3V4 Vancouver, BC, Canada; 8 Department of Genetics, Children's Hospital of Eastern Ontario, 401 Smyth Road, Ottawa, Ontario, K1H 8L1, Canada; 9 Department of Ophthalmology, Children's Hospital Boston, 300 Longwood Ave, Boston, MA, 02115, USA; 10 Khatam Eye Hospital, Mashad University of Medical Sciences, Shahid Ghareni Boulevard, Mashhad, Iran; 11 Department of Pediatric Ophthalmology and The Center for Genetic Eye Diseases, Cole Eye Institute, Cleveland Clinic Foundation, i32, 9500 Euclid Avenue Cleveland, OH, 44195, USA; 12 Department of Ophthalmology, University Medical Center Groningen, University of Groningen, PO Box 30001, Groningen, 9700RB, The Netherlands; 13 Department of Neurology, Royal Devon and Exeter Hospital, Barrack Road, Exeter, Devon, EX2 5DW, UK; 14 Peninsula Medical School, Barrack Road, Exeter EX2 5DW, UK; 15 Department of Neurology, Martin-Luther University Halle -Wittenberg, Ernst-Grube-Str. 40, D-06097 Halle/Saale, Germany; 16 Strabismus Unit, SB Ulucanlar Eye Hospital, Kuzgun sok. 48/3 A, Ayranci, Ankara, Turkey; 17 Department of Pediatric Ophthalmology, Wills Eye Hospital, 900 Walnut St Philadelphia, PA 19107, USA

## Abstract

**Background:**

Congenital fibrosis of the extraocular muscles types 1 and 3 (CFEOM1/CFEOM3) are autosomal dominant strabismus disorders that appear to result from maldevelopment of ocular nuclei and nerves. We previously reported that most individuals with CFEOM1 and rare individuals with CFEOM3 harbor heterozygous mutations in *KIF21A*. *KIF21A *encodes a kinesin motor involved in anterograde axonal transport, and the familial and *de novo *mutations reported to date predictably alter one of only a few KIF21A amino acids – three within the third coiled-coil region of the stalk and one in the distal motor domain, suggesting they result in altered KIF21A function. To further define the spectrum of *KIF21A *mutations in CFEOM we have now identified all CFEOM probands newly enrolled in our study and determined if they harbor mutations in *KIF21A*.

**Results:**

Sixteen CFEOM1 and 29 CFEOM3 probands were studied. Three previously unreported *de novo *KIF21A mutations were identified in three CFEOM1 probands, all located in the same coiled-coil region of the stalk that contains all but one of the previously reported mutations. Eight additional CFEOM1 probands harbored three of the mutations previously reported in *KIF21A*; seven had one of the two most common mutations, while one harbored the mutation in the distal motor domain. No mutation was detected in 5 CFEOM1 or any CFEOM3 probands.

**Conclusion:**

Analysis of sixteen CFEOM1 probands revealed three novel *KIF21A *mutations and confirmed three reported mutations, bringing the total number of reported *KIF21A *mutations in CFEOM1 to 11 mutations among 70 mutation positive probands. All three new mutations alter amino acids in heptad repeats within the third coiled-coil region of the KIF21A stalk, further highlighting the importance of alterations in this domain in the etiology of CFEOM1.

## Background

Congenital fibrosis of the extraocular muscles type 1 (CFEOM1; MIM#135700) is a congenital eye movement disorder inherited as an autosomal dominant trait and characterized by bilateral ptosis and bilateral ophthalmoplegia with the globes fixed downward and unable to be raised above the horizontal midline. Affected individuals have dysplasia of the levator palpebrae superioris and superior rectus muscles that elevate the eyelid and globe, respectively, absence of the superior division of the oculomotor nerve that innervates these muscles, and loss of corresponding motoneurons in the midbrain oculomotor nucleus [1,2]. CFEOM3 (MIM#600638 & MIM#607034) is a similar disorder with a somewhat broader phenotype; in pedigrees with autosomal dominant CFEOM3, some individuals may have the characteristic clinical features of CFEOM1, but at least one affected family member does not meet CFEOM1 criteria. S/he may be unilaterally affected, have one or both globes fixed at or above the midline, or have residual upgaze [3-5].

Most individuals with CFEOM1 and rare individuals with CFEOM3 harbor heterozygous mutations in the developmental kinesin, *KIF21A *[6-8]. Kinesins (KIFs) are molecular motors that transport cargo along microtubules and, in neurons, are responsible for anterograde axonal transport. In mouse development, *Kif21a *expression begins ~E10.5, the time at which the oculomotor nerve and extraocular muscles are developing [6], and is expressed in neurons [9]. The KIF21A cargo is not known.

The 38 exons of *KIF21A *encode a 1674 amino acid protein. Remarkably, only 8 unique mutations altering only 4 amino acids have been identified among the 56 CFEOM1 and 3 CFEOM3 probands harboring *KIF21A *mutations reported to date by us [6-8] and by others [13-16]. Three of these amino acid residues are located in heptad repeats within the third coiled-coil region of the KIF21A stalk, and one of these, R954, is altered in ≥ 86% of all individuals with *KIF21A *mutations reported to date. Two unrelated probands with CFEOM1 harbor a mutation that alters the fourth residue, located at the end of the motor domain. To further expand the definition of *KIF21A *mutations that result in CFEOM, we now report the mutations we have identified in CFEOM probands who were enrolled in our study since our previous mutation reports [6-8].

## Results

**Figure 1:**
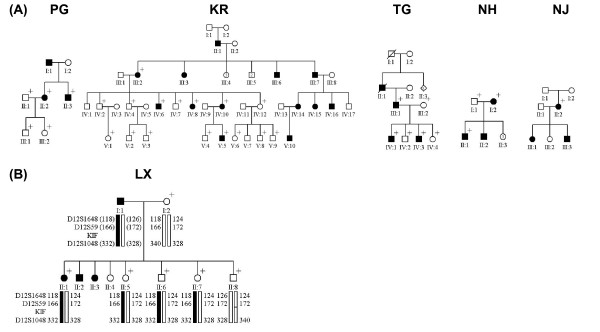
**Pedigrees with autosomal dominant inheritance of CFEOM1**. Black circles/squares indicate clinically affected individuals, and plus signs denote individuals who participated in this study. **(A) **Pedigrees harboring *KIF21A *mutations: pedigrees KR and TG harbor 2860C>T; pedigrees NH and NJ harbor 2861G>A; pedigree PG harbors 1067T>C. **(B) **Haplotype analysis of pedigree LX at the *KIF21A *locus. Genotyping data and schematic segregating haplotype bars for chromosome 12cen markers are shown below the symbol for each participant. A black bar indicates the haplotype passed from the affected father to his affected daughter. This haplotype is also inherited by three of the four unaffected siblings who participated in the study. A *KIF21A *mutation was not detected in this pedigree.

Since our previous reports of *KIF21A *mutations in CFEOM [6-8], we have enrolled an additional 16 CFEOM1 probands of varying ethnicities, six of whom segregate CFEOM1 within their family as a dominant trait (Table 1, Fig. 1A). We have also enrolled 13 familial and 16 sporadic CFEOM3 probands.

**Table 1:** Summary of CFEOM1 probands included in report.

**Pedigree**	**Inheritance**	**Ethnicity**	**KIF21A mutation**	***De novo***	**KIF21A linkage**	**FEOM3 linkage**	**Additional Features**
PG	AD	Caucasian	1067T>C		c/w linkage		
KR	AD	Hispanic	2860C>T		linked		
TG	AD	Caucasian	2860C>T		c/w linkage		
NH	AD	Turkish	2861G>A				
NJ	AD	Caucasian	2861G>A				
LX	AD	Iranian	None		Reduced penetrance	Reduced penetrance	
RY	Sporadic	Caucasian	2830G>C	Yes			+
IW	Sporadic	Caucasian	2860C>T	Yes			
JT	Sporadic	Caucasian	2860C>T				
IP	Sporadic	Caucasian	2860C>T				
SK	Sporadic	Caucasian	2861G>T	Yes			+
XF	Sporadic	Caucasian	3022G>C	Yes			
NG	Sporadic	Turkish	None				
RZ	Sporadic	African	None				+
RX	Sporadic	Caucasian	None				
SY	Sporadic	Caucasian	None				

c/w linkage = consistent with linkage; Reduced penetrance = consistent with linkage with reduced penetrance.

The affected members of all of the pedigrees that segregate CFEOM1 as a dominant trait have classic CFEOM1 without additional features. Three probands with sporadic CFEOM1, however, have additional clinical features. RY has evidence of aberrant innervation with elevation of the left eye and adduction of the right eye while chewing, and SK has bilateral Marcus Gunn jaw winking phenomenon and pupils that respond sluggishly to light. These additional features are similar to the aberrant eye movements and Marcus Gunn phenomenon we have reported previously in a subset of CFEOM1 patients harboring *KIF21A *mutations [8,17]. Together with our MR imaging data [2], this clinical data has supported the presence of widespread dysinnervation in a subset of CFEOM1 patients who harbor *KIF21A *mutations. Individual RZ, however, has facial weakness and brain MR imaging that reveals mild thinning of the splenium of the corpus collosum, features that have not been associated with *KIF21A *mutations.

We identified heterozygous *KIF21A *mutations in 11 of the 16 CFEOM1 probands, and none of the CFEOM3 probands. Eight of the CFEOM1 probands harbor previously reported *KIF21A *mutations: 5 harbor the most common CFEOM1 mutation, 2860C>T, which alters the first nucleotide of the triplet codon encoding amino acid residue 954 (R954W); 2 harbor the second most common mutation, 2861G>A, which alters the second nucleotide of the same triplet codon (R954Q); while 1 harbors the 1067T>C mutation which alters a residue located at the end of the kinesin motor domain (M356T), and is the third proband with this mutation we have identified. In all familial cases, the mutations co-segregated with CFEOM1 and were absent in unaffected family members. In the one case of sporadic CFEOM1 for which we had the participation of both parents (pedigree IW), the mutation was *de novo *(Table 1).

**Figure 2:**
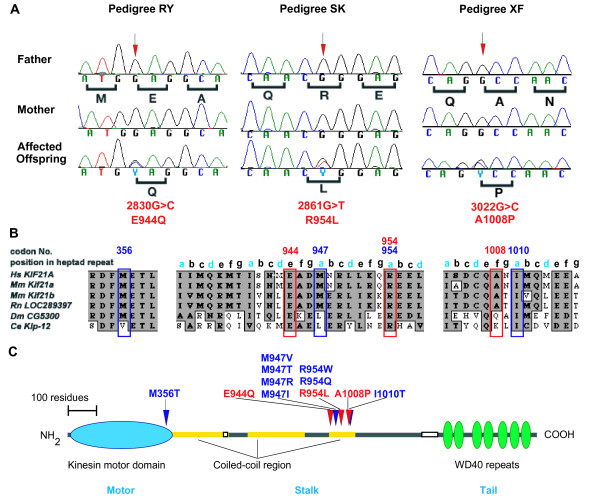
**Nucleotide sequence, amino acid positions, and conservation of the three new *KIF21A *mutations**. (A) Heterozygous *de novo KIF21A *mutations in probands of pedigrees RY, SK and XF. Sequence chromatographs of the unaffected parents are normal (top 2 rows), while sequence chromatographs of the affected offspring with CFEOM1 (bottom row) each reveals a heterozygous *KIF21A *mutation. The normal sequence and corresponding amino acid residues are indicated under each father's sequence chromatograph (black), while the mutation and resulting amino acid substitution are denoted under each affected proband's sequence (red). (B) Portions of the human KIF21A amino acid sequence that surround each of the reported CFEOM1 mutations aligned with homologous/paralogous sequence. Positions a-g of heptad repeat sequence are denoted. Identical amino acid residues are highlighted in dark gray; similar residues are highlighted in light grey. The residues altered by the three new mutations are boxed in red, and the previously reported mutations are boxed in blue. (C) Predicted KIF21A protein structure. The amino acid residues altered by the 3 new heterozygous mutations are depicted in red with red arrows above the protein pointing to their predicted positions. The 8 previously reported *KIF21A *mutations are indicated in blue and their locations are indicated by blue arrows above the protein.

Three previously unreported *KIF21A *mutations were identified in three individuals with sporadic CFEOM1. Probands from pedigrees SK, RY, and XF harbor *de novo *heterozygous *KIF21A *missense mutations 2861G>A (R954L), 2830G>C (E944Q), and 3022G>C (A1008P), respectively (Fig. 2A). These three mutations were not found in the parents of the probands (Fig. 2A), or in 162 control chromosomes of mixed ethnicity. All three mutations are predicted to result in nonconservative amino acid substitutions. Residues R954 and E944 are conserved in mouse, rat, *D. melanogaster*, and *C. elegans*, while A1008 is conserved between human, mouse and rat (Fig. 2B).

## Discussion

We have re-confirmed three of the reported 'hotspot' CFEOM1 mutations in *KIF21A *in eight additional probands, and have identified three previously unreported heterozygous *de novo KIF21A *mutations in three individuals with sporadic CFEOM1. Combining this with the published data, all 11 *KIF21A *mutations identified to date in CFEOM probands are missense mutations, and are predicted to result in the alteration of only 6 of the 1674 amino acid residues that make up the KIF21A protein (summarized in Table 2). All 11 nucleotides and all 6 altered amino acid residues are identical in mouse and human, and the amino acid residues are highly conserved in other species. Moreover, 10 of the 11 mutations, including all 3 new mutations presented in this report, alter amino acids in the third coiled-coil region of the KIF21A stalk (Fig. 2C). The 2861G>T substitution found in pedigree SK becomes the third CFEOM1 mutation to alter KIF21A residue R954, and mutations of this triplet codon currently account for 84% of *KIF21A *mutations in CFEOM. In contrast, 4 of the 11 mutations alter residue M947 but account for only 6% of the total reported mutations.

**Table 2:** Summary of *KIF21A *mutations reported in CFEOM probands to date.

Phenotype & Mutation	Amino acid	Previous Engle Lab Reports^6–8^	Other Lab's Reports^13–16^	Current Report	Total	% mutations
**CFEOM1 Total**		**45**	**12**	**11**	**68/70**	**97%**

1067 T>C	M356T	2	0	1	3	4.0%
2830G> C	E944Q	0	0	1	1	1.5%
2839A>G	M947V	1	0	0	1	1.5%
2840T>C	M947T	1	0	0	1	1.5%
2840T>G	M947R	1	0	0	1	1.5%
2860C>T	R954W	32	12	5	49	70%
2861G>A	R954Q	6	0	2	8	11%
2861G>T	R954L	0	0	1	1	1.5%
3022G>C	A1008P	0	0	1	1	1.5%
3029T>C	I1010T	2	0	0	2	3.0%

**CFEOM3 Total**		**2**	**0**	**0**	**2/70**	**3%**

2841G>A	M947I	1	0	0	1	1.5%
2860C>T	R954W	1	0	0	1	1.5%

The structure of the KIF21A protein includes an N-terminal motor domain, a central stalk, and C-terminal tail domain (Fig. 2C). The motor domain contains the binding site for the microtubule, and tends to be highly conserved. The tail domain diverges among KIFs, and typically contains the site where cargo is loaded, often via an adaptor/scaffolding protein or protein complex [10]. The stalk domain is a flexible connector between the motor and tail that contains α-helical coiled-coil repeats. Typically, the stalk repeats near the motor domain of kinesins are the site of KIF homo- or heterodimerization, permitting two KIF motors to 'walk' down the microtubule. In addition, in some KIFs the coiled-coil domains toward the C-terminal end of the stalk have been demonstrated to interact with cargo [11,12].

The amino acids in the coiled-coil region of the stalk form heptad repeats. Within the repeated heptad consensus sequence, the first (a) and fourth (d) positions are generally nonpolar or hydrophobic amino acids, and when the coiled-coil regions of two proteins interact, these positions are typically internalized and stabilize the dimeric structure. The three amino acid residues in the KIF21A stalk previously reported to be altered in CFEOM (M947, R954, I1010) are each located at the (a) position of heptad repeats within the coiled-coil domain (Fig. 2C), and may normally modulate the assembly and stability properties of the protein structure and interaction. The novel mutation, R954L, identified in SK and the recurrent mutations R954W and R954Q identified in seven probands in this study strengthen our hypothesis that the highly conserved and positively charged arginine residue R954 is an important site for KIF21A function. Two of the new mutations, 2830G>C and 3022G>C, however, are the first CFEOM mutations within the coiled-coil domain that alter amino acids not in the (a) position of heptad repeats, altering instead residues in the (e) and (f) positions, respectively (Fig. 2C). These remaining positions (b, c, e, f, g) in the heptad repeat are generally exposed on the surface of the protein, and these new mutations demonstrate that their disruption can also result in the CFEOM1 phenotype.

The specificity of CFEOM mutations for particular residues within one C-terminal coiled-coil domain of the KIF21A stalk suggests that these mutations alter a critical and specific function of this domain [18,19]. They may alter dimerization between *KIF21A *and itself or another kinesin, may interfere with binding of a specific cargo complex, or may result in binding of an alternative partner. In any of these cases, the likely result is failure of delivery of a cargo that is important to the development of the oculomotor axis.

We sequenced all remaining *KIF21A *exons and intron-exon boundaries in one autosomal dominant CFEOM1 pedigree (LX), four sporadic CFEOM1 probands (NG, RZ, RX, and SY), and all of the CFEOM3 probands, and did not identify any *KIF21A *mutations. Co-segregation analysis of the CFEOM1 phenotype in pedigree LX with markers at the FEOM1, FEOM2, and FEOM3 loci was then investigated. Although analysis was limited by the enrollment of only one of the three affected siblings, this affected sibling shares the same paternal allele with three of his unaffected siblings at both the FEOM1 and FEOM3 loci (Fig. 1B). Reduced penetrance has not been reported for a CFEOM1 pedigree harboring a *KIF21A *mutation, supporting our hypothesis that this pedigree harbors a disease-causing mutation in a different gene. Finally, to rule out compound heterozygous mutations in *PHOX2A*, the proband was screened for mutations and none were identified (data not shown). The 4 sporadic CFEOM1 probands without mutations may harbor mutations at the FEOM3 locus on chromosome 16, as we have identified rare CFEOM1 pedigrees that map to this locus [6,20]. Alternatively, they could harbor mutations in the promoter of KIF21A or in another unidentified CFEOM gene. Finally, we did not identify mutations in any of the CFEOM3 probands, demonstrating again that *KIF21A *mutations are a rare cause of this form of CFEOM. Given the similarities of the CFEOM1 and CFEOM3 phenotypes and the partial overlap in their genetic etiologies, one can hypothesize that the unidentified FEOM3 gene on chromosome 16 may interact with KIF21A and/or function in the same developmental pathway.

## Conclusion

Analysis of sixteen CFEOM1 probands revealed three novel *KIF21A *mutations and confirmed three reported mutations, bringing the total number of reported *KIF21A *mutations in CFEOM1 to 11 mutations among 70 mutation positive probands. All three new mutations alter amino acids in heptad repeats within the third coiled-coil region of the KIF21A stalk, further highlighting the importance of alterations in this domain in the etiology of CFEOM1. Absence of *KIF21A *mutations in some patients with CFEOM1 confirms the genetic heterogeneity of this clinical phenotype.

## Methods

For this study, we identified in our database all CFEOM probands ascertained since our previous reports [6-8]. All affected individuals and participating family members were enrolled in our ongoing research study of congenital eye movement disorders approved by the Children's Hospital Boston Institutional Review Board. Informed consent was obtained after explanation of the nature and possible consequences of the study, and each participant donated a blood or saliva sample from which genomic DNA was extracted using a standard protocol. The research followed the tenets of the Declaration of Helsinki.

High-molecular weight genomic DNA was extracted from blood and salivary samples using the Puregene kit (Gentra), and the purifier solution kit (DNA Genotek), respectively. *KIF21A *exons 8, 20 and 21 and their exon-intron boundaries were first amplified from genomic DNA of each proband using the polymerase chain reaction (PCR) and HotStar Taq DNA polymerase (Qiagen, Germany), as these three exons contain all CFEOM1 mutations reported to date. The resulting amplicons were then directly sequenced on an ABI 3730 Sequence Analyzer (Applied Biosystems, Foster City, CA). If a mutation was not identified within one of these three *KIF21A *exons, the remaining 35 *KIF21A *coding exons were amplified and each amplicon analyzed by denaturing high-performance liquid chromatography (DHPLC, Transgenomic, Inc., Omaha, NE) and/or direct sequencing. When a sequence variant was identified in the proband of an autosomal dominant pedigree, its co-segregation within the family was confirmed. When a sequence variant was identified in a sporadic case, parental DNA was amplified and sequenced to determine if the mutation were *de novo*. Primer sequences and PCR conditions are published [6].

To determine if a previously unreported sequence variation was a polymorphism, SNP databases were interrogated and each variation was looked for in a control panel of 81 DNA samples (162 chromosomes) of mixed ethnicity. PCR amplicons that included the variation were analyzed by either DHPLC using appropriate denaturing temperatures and acetonitrile gradients, or by size analysis following restriction digest by *Dra*III (New England BioLabs, Ipswich, MA). DHPLC conditions and restriction digestion condition are available on request. For any pedigree that segregated CFEOM1 in more than one family member and in which a *KIF21A *mutation was not identified, co-segregation analysis was performed between the CFEOM1 phenotype and fluorescently labeled microsatellite markers spanning each of the three FEOM loci: FEOM1 locus (D12S1648, D12S1692, D12S59, D12S1067, D12S1048); FEOM2 locus (D11S4162, D11S1314, D11S1369); and FEOM3 locus (D16S498, D16S2621, D16S3121, D16S303). Fluorescently labeled primers were purchased from Invitrogen, and amplicons were generated by 30 cycles of PCR amplification containing 10–30 ng of genomic DNA in 5-μl reaction volumes of Qiagen's Taq PCR Master Mix containing 2 pmol of each fluorescent primer pair, 1 nmol each of dATP, dTTP, dGTP, and dCTP, and 0.15 U Taq polymerase. The products were analyzed in an Applied Biosystems 3730 DNA Analyzer. **GenBank accession numbers**. Human *KIF21A *cDNA reference sequence is AY368076; human *KIF21A *genomic reference sequences are AC084373, AC090668 and AC121334; Mouse *Kif21a *cDNA sequence is NM_016705 protein is NP_057914; Mouse *Kif21b *protein sequence is NP_064346; Rat Kif21 protein sequence is XP_223090; *Drosophila melanogaster *Kif21 protein sequence is NP_609398; *Caenorhabditis elegans *Klp-12 protein sequence is BAB18763.

## Abbreviations

CFEOM1 = Congenital fibrosis of the extraocular muscles type 1

CFEOM3 = Congenital fibrosis of the extraocular muscles type 3

PCR = polymerase chain reaction

DHPLC = denaturing high-performance liquid chromatography

SNP = single nucleotide polymorphism

## Authors' contributions

W-MC performed KIF21a mutation screening in all and undertook the control screening for all the novel mutations identified in this manuscript. WC also wrote the first draft of the manuscript and prepared figure 2.

CA collected all the samples for this study, performed linkage analysis on our familial pedigrees, edited the manuscript, prepared all the tables and figure 1 in the manuscript.

ECE was the PI for this study. She conceived of the project, oversaw all aspects of the study, reviewed all of the data, edited the manuscript, and provided the funding.

EY performed KIF21a mutation screening in one family.

The remaining authors each contributed to the paper by ascertaining the pedigrees, enrolling the participants, and obtaining and helping us in the interpretation of all clinical phenotyping data. All authors read and approved the final manuscript.
